# Oxidative Stress Signaling Mediated Pathogenesis of Diabetic Cardiomyopathy

**DOI:** 10.1155/2022/5913374

**Published:** 2022-01-22

**Authors:** Zhaobing Tang, Peng Wang, Chao Dong, Juan Zhang, Xiong Wang, Haifeng Pei

**Affiliations:** ^1^Department of Rehabilitation Medicine, The General Hospital of Western Theater Command, Chengdu 610083, China; ^2^Department of Cardiology, The General Hospital of Western Theater Command, Chengdu 610083, China; ^3^Emei Rehabilitation and Sanatorium Center of PLA, Leshan 614201, China

## Abstract

As a serious cardiovascular complication, diabetic cardiomyopathy (DCM) refers to diabetes-related changes in myocardial structure and function, which is obviously different from those cardiomyopathy secondary to hypertension, coronary heart disease, and valvular disease. The clinical features of DCM are left ventricular hypertrophy, myocardial fibrosis, and impaired diastolic function. DCM will lead to cardiac dysfunction, eventually progress to cardiac arrhythmia, heart failure, and sudden cardiac death. At present, the pathogenesis of DCM is complex and not fully elucidated, and oxidative stress (OS), inflammatory response, glucolipid metabolism disorder, etc., are considered as the potential pathophysiological mechanisms. As a consequence, there is no specific and effective treatment for DCM. OS refers to the imbalance between reactive oxygen species (ROS) accumulation and scavenging, oxidation, and antioxidants in vivo, which is widely studied in DCM. Numerous studies have pointed out that regulating the OS signaling pathways and reducing the generation and accumulation of ROS are potential directions for the treatment of DCM. This review summarizes the major OS signaling pathways that are related to the pathogenesis of DCM, providing ideas about further research and therapy.

## 1. Introduction

Diabetes is a chronic metabolic disease that threatens the health of hundreds of millions. The incidence of diabetes was 4.6% in 2000 and quickly increased to 10.5% in 2021, and alarmingly, the cases are predicted to reach 783 million by 2045 [1]. Of all the cases, over 90% are type 2 diabetes mellitus (T2DM), which is responsible for more than 6 million deaths in 2021 [1]. Diabetic cardiovascular diseases (DCVDs) are the leading causes of death in diabetic patients, accounting for more than 50% [2]. DCVD includes coronary heart disease, cerebrovascular disease, congestive heart failure, and diabetic cardiomyopathy (DCM) [3, 4]. As a serious type of DCVD, DCM is defined as a specific disease with cardiac structural abnormalities and dysfunctions in diabetic patients independent of uncontrolled hypertension, coronary artery disease, significant valvular disease, and congenital heart disease [5, 6], but there is still no universally accepted definition and no authoritative epidemiological data on morbidity and mortality [7]. Its histopathology reveals that the main pathological features are myocardial hypertrophy and fibrosis [8, 9], which exacerbate cardiac hypertrophy, reduce myocardial compliance, cause cardiac diastolic dysfunction [3, 10], and eventually lead to cardiac arrhythmia, heart failure or sudden cardiac death [11, 12]. The pathophysiology of DCM is not fully elucidated. Previous studies have shown that (1) increased flux of glucose and other sugars through the polyol pathway, (2) increased intracellular formation of advanced glycation end products (AGEs), (3) increased expression of the receptor for AGEs and its activating ligands, (4) activation of protein kinase (PK) C isoforms, and (5) overactivity of the hexosamine pathway are probably the 5 major mechanisms for tissue damage in DCM [13]. The excessive production of mitochondrial reactive oxygen species (ROS) is a single upstream event by which all those 5 mechanisms are activated [14]. Therefore, ROS plays critical roles in the progression of DCM.

Oxygen free radicals, together with nonradicals, constitute ROS [15], which is salutary when it is a physiological dose. Oxidative stress (OS) refers to the accumulation of ROS or the attenuation of antioxidant mechanisms in cells or the imbalance between oxidation and antioxidation in vivo, which finally leads to the damage of biomacromolecules [16, 17]. In present paper, we focus on recent studies and summarize the OS-related signaling pathways associated with DCM.

## 2. ROS Generation and Its Role in DCM Progression

Mitochondria are the major sites of energy generation. It is estimated that mitochondria in the heart produce up to 6 kg of ATP per day [18]. In addition to ATP production, mitochondria are involved in the process of OS, which is related to the ROS production and ROS scavenging. The ROS production owe to the electron transport chain, a main generator of mitochondrial free radicals, which is located in the inner mitochondrial membrane [19, 20]. Apart from mitochondria, the NAD(P)H oxidase, xanthine oxidase, and uncoupled nitric oxide synthase are also the source of ROS [10]. There are small amounts of ROS in the heart of physiological conditions, and the basal levels of ROS are essential for maintaining various cellular functions [21]. When the oxidative homeostasis of heart is broken, mitochondrial respiration is impaired, mitochondrial ROS production exceeds its clear capacity, and ROS accumulation occurs; free radicals significantly increase and damage the myocardium [22, 23]. Conversely, excessive ROS can cause oxidative damage to mitochondrial DNA, proteins, and membranes, leading to mitochondrial dysfunction and contributing to multiple diseases [24]. In diabetics, by increasing glucose oxidation in the citric acid cycle, hyperglycemia itself can increase hyperlipidemia or insulin resistance and lead to OS directly or indirectly [25]. Insulin resistance or insulin deficiency leads to hyperglycemia, which contributes to increase ROS production [26, 27]. Excessive ROS generation results in cardiac OS and inflammation, which leads to cardiac fibrosis, cardiac hypertrophy, coronary microvascular impairment, and left ventricular diastolic dysfunction. Usually, ventricular systolic dysfunction occurs in the late stage and eventually develops into heart failure [5, 28, 29]. But their detailed pathological mechanisms are still unclear. DCM is a term used to describe diabetes-related changes in myocardial structure and function and to distinguish these changes caused by hypertension or coronary artery disease [30]. Its characters are hypertrophie concentrique, myocardial fibrosis, cardiac stiffness, and impaired diastolic function [3, 10].

## 3. Oxidative Stress Signalings Involved in the Pathogenesis of DCM

Several mechanisms are involved in the pathogenesis of DCM, among which OS plays a causative role in DCM pathophysiology. However, how OS promotes the development of DCM is incompletely clear. We summarized some relevant studies in recent years.

### 3.1. Nrf2-Mediated Antioxidant Signaling Pathways

Nuclear factor erythroid 2-related factor 2 (Nrf2) is a transcription factor, which binds to its repressor Kelch-like epichlorohydrin-associated protein 1 (Keap1) and exists in the cytoplasm. The conjugate of Nrf2 and its repressor Keap1 can be dissociated and activated by OS; then, Nrf2 translocates into the nucleus to induce the expression of antioxidant genes. Meanwhile, among the downstream Nrf2-driven genes, p62 is a specific autophagy receptor of Keap1, which promotes Keap1 degradation and activates Nrf2 [31]. The dynamic interaction between Nrf2, Keap1, and p62 is to maintain cardiac homeostasis and reduce damage during OS in diabetics. The feed-forward loop linking Nrf2-Keap1-p62 is broken in streptozotocin- (STZ-) induced diabetic rats. After allopurinol treatment, it significantly increases the expression of Nrf2 and p62 and reduces the expression of Keap1 to repair the circuit and ease OS and apoptosis induced by high glucose [32]. Normally, Nrf2 activity is suppressed by its native repressor Keap1; however, OS or electrophilic stress liberates Nrf2 from Keap1, allowing Nrf2 to translocate into the nucleus and bind to the promoter region of the antioxidant response element (ARE). This activates antioxidant genes such as glutathione S transferase, SOD, and quinone oxidoreductase 1 (NQO-1) [33]. These antioxidant enzymes alleviate OS damage by enhancing antioxidant capacity, inhibiting inflammation, and transporting toxic metabolites. Therefore, the Nrf2-Keap1-ARE pathway is identified as the major mechanism of myocardial defense against oxidative damage in diabetes mellitus and high glucose [33]. Empagliflozin, a sodium-glucose co-transporter 2 (SGLT2) inhibitor, is a novel oral hypoglycemic drug to reduce hyperglycemia by highly selective inhibition of SGLT2 [34]. Empagliflozin attenuates OS by promoting the nuclear translocation of Nrf2 and activating the Nrf2-ARE pathway, improving antioxidant levels and reducing oxidative products in the type 2 diabetic KK-Ay mice model [35], which remarkably reduces heart failure and cardiovascular-related deaths in diabetic patients [34]. The activation of Nrf2 increases mRNA levels of Nrf2-target genes Heme oxygenase-1 (HO-1) and NQO-1 to alleviate OS and prevent cardiac dysfunction in STZ-induced diabetic mice [36]. Allisartan isoproxil, a blocker of the angiotensin II receptor that is used to reduce the risk of heart disease, mitigates diabetes-induced OS and inflammation by upregulating Sirt1/Nrf2/HO-1 signal and inhibiting the NF-*κ*B activation, respectively, in diabetic cardiomyopathy (DCM) rats [37]. Another study also confirmed that increasing the expression of Nrf2/HO-1 signaling pathway can alleviate the OS in DCM [38]. Nrf2/HO-1 can also alleviate inflammation by restraining nuclear translocation of RelA (p65), a canonical NF-*κ*B subunit. In reverse, Nrf2 can be inhibited by p65 activity, and its mechanism is a competition for the transcriptional coactivator CREB-binding protein- (CBP-) p300 complex [39]. Some natural products such as garlic, curcumin, sulforaphane, and phenolic acids affect the Nrf2 activation to alleviate OS and maintain cellular antioxidant protection and redox homeostasis, achieving cardiac protection [40]. Various substances protect diabetic cardiomyocyte against OS and inflammation-induced injury through activating adenosine monophosphate-activated protein kinase (AMPK)/Nrf2/HO-1 signaling pathway [41–43]. In general, Nrf2 acts as a mediator between upstream genes and downstream target genes in multiple signaling pathways, which plays an antioxidant role in DCM to alleviate OS damage and improve cardiac function.

### 3.2. NF-*κ*B-Mediated Proinflammation Signaling Pathways

Nuclear factor-*κ*B (NF-*κ*B) is a family of transcription factors, which is secluded by inhibitor of NF-*κ*B (I*κ*B), known as a family of inhibitory proteins, in the cytoplasm. The I*κ*B kinase (IKK) complex leads to I*κ*B phosphorylation, ubiquitination, and degradation and releases NF-*κ*B translocation into the nucleus to activate inflammatory gene expression [44, 45]. Inhibition of IKK partially normalizes ROS levels in hypothalamic arcuate nucleus (ARC) and heart in diabetic rats and alleviates OS. This effect is thought to be related to the suppression of NF-*κ*B signaling pathway [46].

Kosuru et al. confirmed that, respectively, inhibiting NF-*κ*B and nucleotide-binding oligomerization domain-like receptor protein 3 (NLRP3) inflammasome through activating AMPK signaling effectively attenuate the cardiac inflammation; however, the further interaction between NF-*κ*B and NLRP3 inflammasome was not revealed [42]. NLRP3 inflammasome is a multiprotein complex assembled by pattern recognition receptor, which is not critical for the innate immune system but also related to the pathogenesis of several inflammatory diseases including diabetes [47]. Further research showed that the inhibition of NF-*κ*B activation can prevent the expression and activation of NLRP3 inflammasome, which can further convert precursor caspase-1 into cl.casp-1 to trigger mature inflammatory factor IL-1*β* and IL-18 release and induce inflammation in diabetic mice [48].

High glucose-induced ROS mediates cardiomyocyte pyroptosis, a form of programmed cell death that accompanies an inflammatory response [49], by activating NLRP3 inflammasome. NF-*κ*B and thioredoxin-interacting protein (TXNIP) are involved in the ROS-induced NLRP3 inflammasome activation [50]. TXNIP, as a member of *α*-arrestin family, is a multifunctional adaptor protein for different signaling pathways and is related to the regulation of OS [51]. AMPK activated by OS via ROS-dependent phosphorylation can downregulate TXNIP to regulate NLRP3 inflammasome activity and mediate pyroptosis [52, 53]. On one hand, exendin-4 is a peptide hormone belonging to the glucagon superfamily, which promotes insulin secretion to control blood sugar [54]. On the other hand, exendin-4, as an antioxidant, can not only directly inhibit the activation of caspase-1 but also indirectly suppress the activation of caspase-1 by downregulating TXNIP to prevent myocardial pyroptosis caused by high glucose [55]. Hyperglycemia can damage mitochondria which resulting in ROS overproduction and activate NLRP3 inflammasome to injure myocardial cell. Gypenoside, an extract of plant Gynostemma pentaphyllum with antioxidation, antilipidemia, and anti-inflammatory effects [56], inhibits this pathway in diabetic mice, reducing IL-1*β* release and attenuating heart damage [57]. Similarly, the overexpression of ALDH2 could suppress the mitochondrial ROS production and inhibit the occurrence of NLRP3 inflammasome expression to protect the H9c2 cardiac cells against hyperglycemia-induced OS and inflammation, thus protecting cardiomyocytes [58].

Luteolin, a natural flavonoid in many plants with anti-inflammatory and antioxidant activities, exerts dual protective effects by inhibiting ROS-mediated activation of NF-*κ*B which can decrease the proinflammatory cytokines IL-6 and TNF-*α* in the heart [36]. Multiple studies have confirmed that hyperglycemia can upregulate NF-*κ*B expression, but by suppressing NF-*κ*B signaling pathway, it can effectively ease cardiac inflammation and OS and improve cardiac dysfunction and remodeling [59–62]. NF-*κ*B modulates the transcription and activity of Nrf2, having positive and negative bidirectional regulation effect on the target gene expression, while the absence of Nrf2 aggravates NF-*κ*B activity, leading to the increase in the production of inflammatory cytokines [39]. NF-*κ*B and Nrf2 interact with each other to take part in diabetes-induced OS and inflammation. To summarize, NF-*κ*B, as a key regulatory molecule, is a link between OS and inflammation. The ROS-induced NF-*κ*B-NLRP3 signaling activation is a crucial inflammatory signaling pathway in DCM.

### 3.3. Sirt1-Mediated Signaling Pathways Prevent OS Damage

Sirtuin1 (Sirt1), a cellular nicotinamide adenine dinucleotide- (NAD+-) dependent deacetylase that catalyses deacetylation of proteins, is the most studied one in seven members of the sirtuin family (Sirt1-Sirt7), especially in cardiovascular diseases [63]. Sirt1 acts as a protector when suffering from cardiovascular disease and other disorders. In both high glucose-cultured H9c2 cardiomyocyte and STZ-induced diabetic mice, Sirt1 expression decrease is observed; meanwhile, its two crucial downstream enzymes SOD and GSH-Px, whose enzymatic activities are observed to decrease, result in a significant increase in ROS production. Activating the expression of Sirt1 can reverse the above signaling pathway to protect DCM against OS damage [64, 65]. Moreover, Sirt1 blocks the expression of proinflammatory genes via inhibiting NF-*κ*B signaling [66]. Overexpressed microRNA-22 can directly bind to the 3′untranslated repeats (3′-UTRs) of its target gene Sirt1 and alleviate diabetic cardiomyopathy OS via upregulating Sirt1 in vivo and in vitro [67]. Through animal and cell experiments, Zhang et al. confirmed that Honokiol, a plant extract from Magnolia grandiflora seed cone, has been widely used in traditional Chinese medicine could attenuate OS and apoptosis by activating Sirt1-Nrf2 signaling pathway, effectively improve myocardial ischemia/reperfusion injury in type 1 diabetic rats, and achieve cardiac protection [68]. Caloric restriction can increase the expression of Sirt1 and peroxisome proliferator-activated receptor-*γ* coactivator-1*α* (PGC-1*α*), improve mitochondrial function, reduce ROS production, relieve cardiac OS and inflammation caused by hyperglycemia, and play a cardioprotective role [69]. Previous studies have also shown that activation of PGC-1*α* reduced mitochondrial ROS in adipocytes via the induction of HO-1 [70]. Recent studies have confirmed that activation of the Sirt1-PGC-1*α* signaling pathway protects against Drp1 (a mitochondrial fission-related proteins)-mediated mitochondrial fission (a dynamic process that maintains mitochondrial homeostasis and regulates cellular stress response) [71], suppresses mitochondrial OS, and improves mitochondrial dysfunction [72]. Therefore, the Sirt1-PGC-1*α*-HO-1 axis plays a key role in protecting diabetic heart against hyperglycemia-induced OS damage [69, 73].

### 3.4. Other Signaling Pathways of OS-Related in DCM

Hyperglycemia leads to OS and excessive ROS production and induces cardiovascular damage. Ren et al. showed that activation of Sirt1-FOXO1 and PI3K-Akt signaling pathways can control blood glucose metabolism, reduce ROS production, inhibit OS and myocardial cell apoptosis, delay cardiac complications, and achieve the purpose of alleviation or even treatment of diabetic cardiomyopathy [74]. Forkhead box protein O1 (FOXO1) is a transcriptional factor involved in regulating myocardial metabolism. Overactivation of FOXO1, through increasing pyruvate dehydrogenase kinase 4 (PDK4) and carnitine palmitoyltransferase 1 (CPT1) expression, induced disarranged cardiac oxidative metabolism. Treatment of diabetic rats with FOXO1-selective inhibitor reduces mitochondrial ROS production, restores mitochondrial morphology and mitochondrial membrane potential, and reverts myocardial apoptosis [75]. In STZ-induced DCM mouse model, the levels of phosphatidylinositol 3-kinase (PI3K) and Akt mRNA expression were increased, which can upregulate the Bax and caspase-3 protein, while the levels of Bcl-2, PI3K, p-GSK-3b/GSK-3b, and p-Akt expression were decreased, and this means the PI3K/Akt signaling pathway is involved in mediating myocardial damage caused by OS and inflammation [76]. Additionally, PI3K/Akt/FoxO3a pathway also has been confirmed to improve cardiac function and prevent cardiac remodeling in diabetic cardiomyopathy by inhibiting apoptosis [77]. Importantly, gestational diabetes mellitus (GDM) exposure can induce OS of cardiac myocyte in offspring and excessive ROS selective activation and increase DNA methyl transferase expression, thereafter leading to DNA hypermethylation, downregulating Sirt1 protein expression and Akt phosphorylation. GDM mediated downregulation of the Sirt1/Akt signaling pathway resulting in aberrant development of heart ischemia-sensitive phenotype in offspring [78].

As a class of highly evolutionally conserved endogenous single-stranded small noncoding RNAs, microRNAs are involved in many biological processes and diseases. miR-30c is the most abundant microRNA in the heart tissue of C57 mice. In db/db mice, the expression of miR-30c decreased significantly and then led to the increase in PGC-1*β*, which enhanced the transcriptional activity of peroxisome proliferator-activated receptor *α* (PPAR*α*). The upregulated PPAR*α* targeted on CD36 and PDK4 genes promotes the uptake of fatty acid and reduces glucose utilization, respectively. This pathway affects mitochondrial basal energy metabolism and produces excessive ROS, eventually leads to myocardial cell apoptosis and cardiac dysfunction, whereas excessive fatty acid intake will further inhibit the expression of miR-30c, forming a vicious cycle. Exogenous miR-30c introduced into db/db mice can play a protective role through the miR-30C/PGC-1*β*/PPAR*α* signaling pathway [79, 80].

Nicotinamide adenine dinucleotide phosphate (NADPH) oxidase (Nox) is a major donor of ROS in myocardial ischemia/reperfusion injury. In vitro and in vivo experiments showed that the OS and programmed cell death were enhanced by inhibiting AMPK pathway, which can activate the subtype Nox2 of NADPH oxidase and exacerbate myocardial infarction size [81]. Through activation of Nox1, TGF-𝛽 mediated fibrosis, NF𝜅B, and ERK1/2 pathways and decreased expression of SOD-1, advanced glycation end product/its receptor (AGE/RAGE) signaling is implicated in OS of diabetes-mediated vascular calcification [82]. Hyperglycemia in diabetic patients induces OS, which leads to subcellular abnormalities such as sarcolemma membrane defects, impairment of sarcoplasmic reticulum function, myofibrillar abnormalities, and abnormal Ca2+-handling of cardiomyocyte, resulting in abnormal cardiac function [28]. Salusin-*β* is a bioactive peptide widely distributed over many tissues, characterized by hemodynamic and mitogenic activity, and can be synthesized in cardiomyocyte locally. Knockout Salusin-*β* can alleviate OS and inflammatory response in DCM via suppressing Nox2/ROS/NF-*κ*B signaling [83].

Mammalian target of rapamycin (mTOR) not only plays a key role in energy metabolism but also takes part in the maintenance of normal microvascular barrier function and endothelial permeability, cardiometabolic homeostasis, and OS. It is activated by ROS and subsequently exerts a dual regulatory role. The chronic increase in mTORC1 activity in T2DM can lead to insulin resistance, resulting in hyperinsulinemia and hyperglycemia [84]. Previous studies showed the activation of its four related signal pathways which are cardioprotective effects: (1) insulin-mediated PI3K/Akt/mTOR signaling pathway, (2) GSK-3*β* inhibition signaling pathway, (3) mTOR-dependent angiogenesis signaling pathway, and (4) mTORC2 activation signaling pathway [85]. Recently, the activation of AMPK which downregulates the mTOR signaling to protect cardiomyocyte against OS and inflammation damage induced by high glucose is demonstrated [86, 87].

## 4. Conclusion

**Figure 1:**
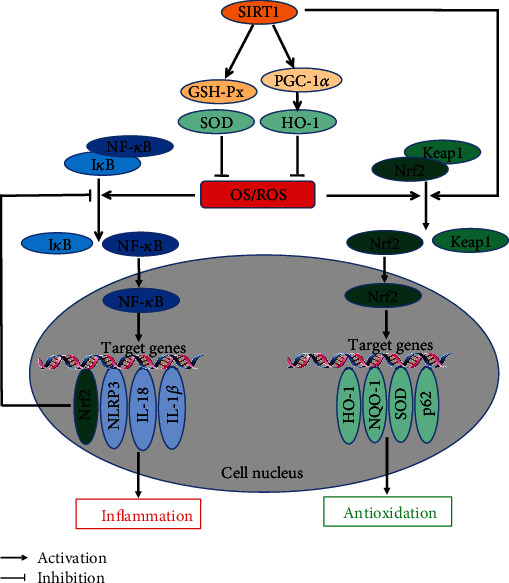
(a) ROS can dissociate the Nrf2/Keap1 complex in the cytoplasm; then, the Nrf2 translocates into the nucleus, acting on downstream antioxidant genes and alleviating OS damage. (b) ROS activates the NF-*κ*B-mediated inflammatory signaling pathway, exacerbating cardiac OS damage. Meanwhile, activated Nrf2 causes a feedback inhibition of NF-*κ*B pathway. (c) Upregulation of SIRT1 suppresses ROS generation and alleviates OS damage through antioxidant enzymes (GSH-Px and SOD) and PCG-1A/HO-1 pathway.

There are various kinds of OS-related signaling pathways involved in the pathogenesis of DCM. Elevated ROS is associated with OS and inflammatory response induced by high glucose in DCM. High glucose inhibits Nrf2 and Sirt1-mediated antioxidant signals and activates NF-*κ*B-mediated proinflammatory signals, respectively. OS and inflammation interact with each other to increase the production of ROS and inflammatory factors, which promote and aggravate cardiac dysfunction and remodeling. Activating the Nrf2 and Sirt1 and inhibiting the NF-*κ*B-mediated signaling pathways can restore the above damage in vivo and in vitro. The above signaling pathways can overlap and influence each other (see Figure 1). Therefore, the pathogenesis of DCM is complex and multifactorial, and further research is needed.
